# Postural stability in individuals with and without motion sickness assessed by computerized posturography: sensory integration and balance mechanisms

**DOI:** 10.1016/j.bjorl.2026.101906

**Published:** 2026-09-22

**Authors:** Arthur Jose Roque Cruz, Paola Piva de Freitas, Danilo Costa Chagas, Raquel Mezzalira, Maria Isabel Ramos do Amaral, Carlos Takahiro Chone

**Affiliations:** a Universidade Estadual de Campinas (UNICAMP), Campinas, SP, Brazil; b Instituto Penido Burnier, Campinas, SP, Brazil

**Keywords:** Motion sickness, Balance, Postural control, Conflict, Habituation

## Abstract

**Objective:**

To evaluate whether individuals with motion sickness present differences in postural control and sensory integration compared with controls, through assessment with computerized dynamic posturography.

**Methods:**

A prospective study was conducted including 30 patients with motion sickness and 61 controls without balance disorders. All participants underwent limit of stability testing and the six conditions of posturography using the Horus® platform. Equilibrium scores and sensory ratios (somatosensory, visual, vestibular, and visual preference) were calculated. Statistical analyses were performed using MannWhitney, chisquare, and ANCOVA tests, adopting a 5% significance level.

**Results:**

After age adjustment, no significant differences were observed in the limit of stability area (p = 0.271) or in equilibrium scores under any of the posturography conditions. Sensory ratios and the composite equilibrium index were also similar between groups.

**Conclusion:**

Individuals with motion sickness exhibited posturographic performance comparable to controls, suggesting preserved sensory integration for balance maintenance outside symptomatic episodes.

## Highlights

• Motion sickness patients had normal posturographic performance.
• No sensory integration differences were found between groups.
• Habituation may explain preserved balance outside symptoms.

## Introduction

Motion sickness is considered a normal physiological response to unfamiliar patterns of movement. Although the pathophysiological mechanism behind symptom onset remains uncertain, the most widely accepted theory is the sensory conflict. This theory posits that motion stimulates the vestibular (semicircular canals and otolith organs), visual, and somatosensory systems. The information from these systems converges in the vestibular nuclei, cerebellum, and cortex, where it is integrated into a common signal. This signal is then compared to previously stored neural memory. If the acquired sensory input is not recognized in comparison to stored information, a conflicting signal is generated that activates the vomiting center and triggers the cascade of symptoms.

The conflict may arise from incongruent information within a single sensory system (e.g., between the semicircular canals and otolith organs) or between multiple systems (e.g., visual-vestibular interaction).^1^

Computerized Dynamic Posturography (CDP) is a tool capable of quantifying the contributions of different systems to postural control using a computerized platform equipped with pressure sensors that detect forces exerted by the soles of the feet. Among various protocols, the Sensory Organization Test (SOT) is the most commonly used, as it quantitatively assesses the use of the three main balance systems ‒ vestibular, visual, and somatosensory ‒ and their integration.^2^

Given that CDP is based on sensory integration, individuals with motion sickness are expected to have greater difficulty maintaining posture under certain test conditions. However, findings in the literature are conflicting. Some authors have reported that motion sickness patients exhibit abnormalities in conditions 4 and 5, which relate to the use of visual and vestibular inputs for balance maintenance, respectively.^3^^,^^4^ Another group observed changes in condition 3, suggesting increased visual dependence due to vestibular hypofunction.^5^ Tal et al. described a shift in strategy during adaptation to visual-vestibular conflict, whereby patients begin to disregard visual input and become more reliant on vestibular and somatosensory cues for postural control.^6^

On the other hand, it is known that when new relationships among sensory inputs are established, the “neural memory” is gradually updated. As a result, the pre-existing postural representation model is progressively modified until the actual and expected sensory information converges, and symptoms no longer occur.^1^^,^^5^ Furthermore, for sensory conflict to take place, the vestibular, visual, and proprioceptive systems must be intact. Under these conditions, it is also reasonable to expect that individuals with motion sickness may exhibit good performance in posturographic assessments.

Understanding the balance strategies used by individuals with motion sickness may offer valuable insights for treatment, particularly through vestibular rehabilitation.

The objective of this study was to investigate whether individuals with and without motion sickness demonstrate functional differences in balance responses during sensory interaction testing.

## Methods

This is a prospective study approved by the Research Ethics Committee (number 2.641.499). All participants were informed about the procedure and signed the Informed Consent Form. The sample consisted of 30 subjects diagnosed with motion sickness according to criteria established by the Bárány Society in 2021^7^ (study group) and 61 adult individuals without complaints related to balance who constituted the control group.

Subjects over 18-years of age with cognitive capacity, visual acuity and postural conditions that allowed posturography to be performed were selected.

All participants had symptoms induced by situations of physical movement (boats, buses, parks, etc.) or visual movement (screens, virtual reality, etc.) whenever exposed to such a situation, with impairment of the activities performed at the time. According to the Bárány Society Guidelines, motion sickness is diagnosed when the sickness inducing stimulus is physical motion of the person and Visually Induced Motion Sickness (VIMS) is diagnosed when the stimulus is visual motion, as shown in Table 1.

**Table 1:** diagnostic criteria for motion sickness and visually induced motion sickness according to the Bárány Society Guidelines.^7^

A) Physical motion of the person or visual motion elicits sign(s) and/or symptom(s) in at least one of the following categories, experienced at greater-than-minimal severity:
1. Nausea and/or gastrointestinal disturbance
2. Thermoregulatory disruption
3. Alterations in arousal
4. Dizziness and/or vertigo
5. Headache and/or ocular strain
B) Sign(s) and/or symptom(s) appear during motion and build as exposure is prolonged
C) Sign(s) and/or symptom(s) eventually stop after cessation of motion
D) Sign(s) and/or symptom(s) are not better accounted for by another disease or disorder

Subjects with sporadic symptoms of motion sickness or that did not compromise activity at the time of the crisis, individuals with eye, rheumatic or orthopedic diseases that did not allow posturography to be performed, or with a history of any vestibular disease, or who were using medications that affect the central nervous system or who did not agree to sign the Informed Consent Form were excluded. Athletes, dancers or any individual potentially presenting balance performance well above average were also excluded.

All participants underwent stability limit determination and six sensory conditions on the Horus® computerized posturography platform (Fig. 1), as previously standardized by our team.^8^

**Fig. 1:**
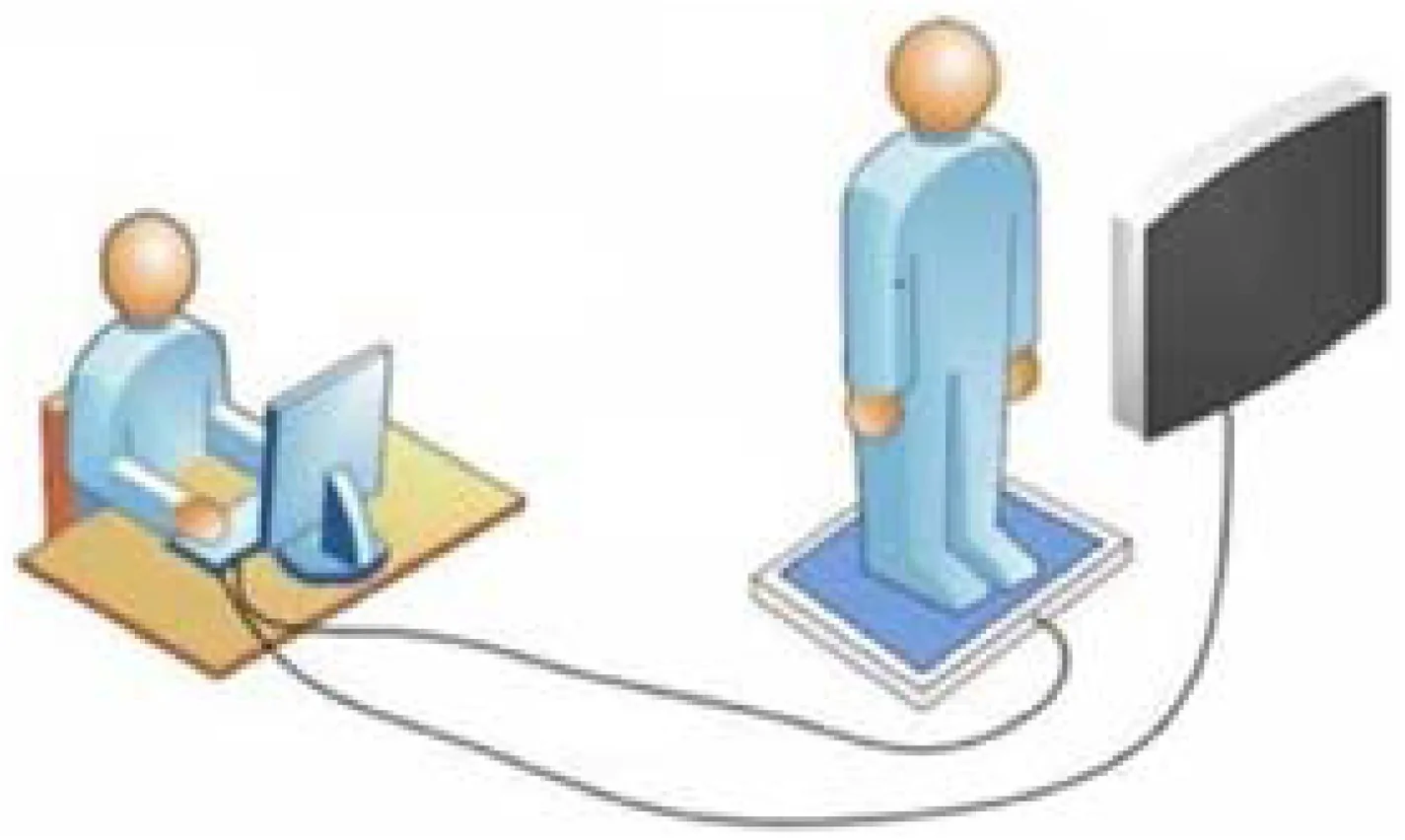
representative image of the equipment and the workstation. (Source: User manual ‒ Horus ‒ System for Posturography and Postural Rehabilitation).

The tests were carried out under the following conditions:

Stability Limit Area (SLA)

Condition 1 (C1) – Fixed reference surface, eyes open, visual surround stationary.

Condition 2 (C2) – fixed reference surface, eyes closed.

Condition 3 (C3) ‒ Fixed reference surface, eyes open, visual surround moving.

Condition 4 (C4) – Unstable reference surface, eyes open, visual surround stationary.

Condition 5 (C5) – Unstable reference surface, eyes closed.

Condition 6 (C6) ‒ Unstable reference surface, eyes open, visual surround moving.

The stable surface corresponds to the platform surface itself, on which the patient stands barefoot and without support, and the unstable surface is tested using a medium-density pad. A 42-inch LED TV set was positioned 1 meter away from the patient’s eyes, on the same horizontal plane of vision, following the manufacturer’s orientation, with the projection of a fixed point and optokinetic tunnel to provide visual conflict. The Stability Limit Area (SLA) and score of equilibrium were analyzed.

Initially, the SLA was marked, respecting the following sequence: forward and return to the starting position; to the right and return to the starting position; to the left and return to the starting position; backward and return to the starting position. Participants were instructed to perform the complete sequence of movements only once, using the ankle strategy, without moving their feet or trunk. If there was any movement of feet or body, the test was restarted. After that, the six conditions were tested for 30 seconds each. Only one record of each condition was made, as allowed by the equipment.

The variables were also analyzed by age and sex and the Equilibrium Score (ES) and Sensory Organization Test (SOT) were calculated for each condition. Quantification of the results obtained ranged from 100% (no displacement recorded by the platform’s sensors) to 0%, which corresponded to a fall in either direction. Based on the ES for each condition, the SOT was performed, consisting in the quantitative analysis of body equilibrium calculated using the ratio between the ES of two conflicting situations, to establish the most requested equilibrium functions in these situations, as described below:

• Somatosensory function: average of condition 2/average of condition 1.
• Visual function: average of condition 4/average of condition 1.
• Vestibular function: average of condition 5/average of condition 1.
• Visual preference: average of condition 3 + 6/average of condition 2 + 5.
• Equilibrium index: mean of conditions 1–6.

The Mann-Whitney, Chi-square e ANCOVA tests were used for comparison between the groups. The level of significance adopted was 5%.

## Results

The study included 30 participants with motion sickness and 61 controls. Most were female (76.7% in the motion sickness group, 62.3% in controls). There was no significant sex-group association. Age differed significantly between groups (p = 0.028). After age adjustment, no significant SLA difference was observed (p = 0.271) (Table 2).

**Table 2:** demographic and clinical profile of study participants.

	Study group (n = 30)	Control group (n = 61)	p-value
Sex			0.257
Female	23 (76.7%)	38 (62.3%)	
Male	7 (23.3%)	23 (37.7%)	
Age	30.6	36.7	**0.028**
SLA (mm^2^)	27483	24501	0.271^a^

a Age-adjusted. SLA, Stability Limit Area.

No significant differences were found between groups in any test condition. Mean values and standard deviations for each condition tested, corrected by the participants ages, are shown in Table 3 and Fig. 2, Fig. 3.

**Table 3:** results of the sensory organization test. significance of differences between groups.

	Study group (n = 30)	Control group (n = 61)	
	Mean ± Standard Deviation	Mean ± Standard Deviation	p-value
Condition 1	99.3 (0.43)	99.3 (0.54)	0.735
Condition 2	99.0 (0.93)	99.1 (0.85)	0.210
Condition 3	99.2 (0.84)	99.3 (0.54)	0.468
Condition 4	99.1 (0.55)	98.7 (0.94)	0.114
Condition 5	98.3 (1.23)	98.0 (1.95)	0.896
Condition 6	98.8 (0.87)	98.9 (0.90)	0.303
Equilibrium index	99.0 (0.60)	98.9 (0.80)	0.718
Somatossensory	99.7 (0.95)	99.8 (0.61)	0.298
Visual	99.8 (0.62)	99.5 (0.83)	0.194
Vestibular	99.0 (1.24)	98.7 (1.82)	0.601
Visual preference	100 (0.48)	98.9 (0.80)	0.718

**Fig. 2:**
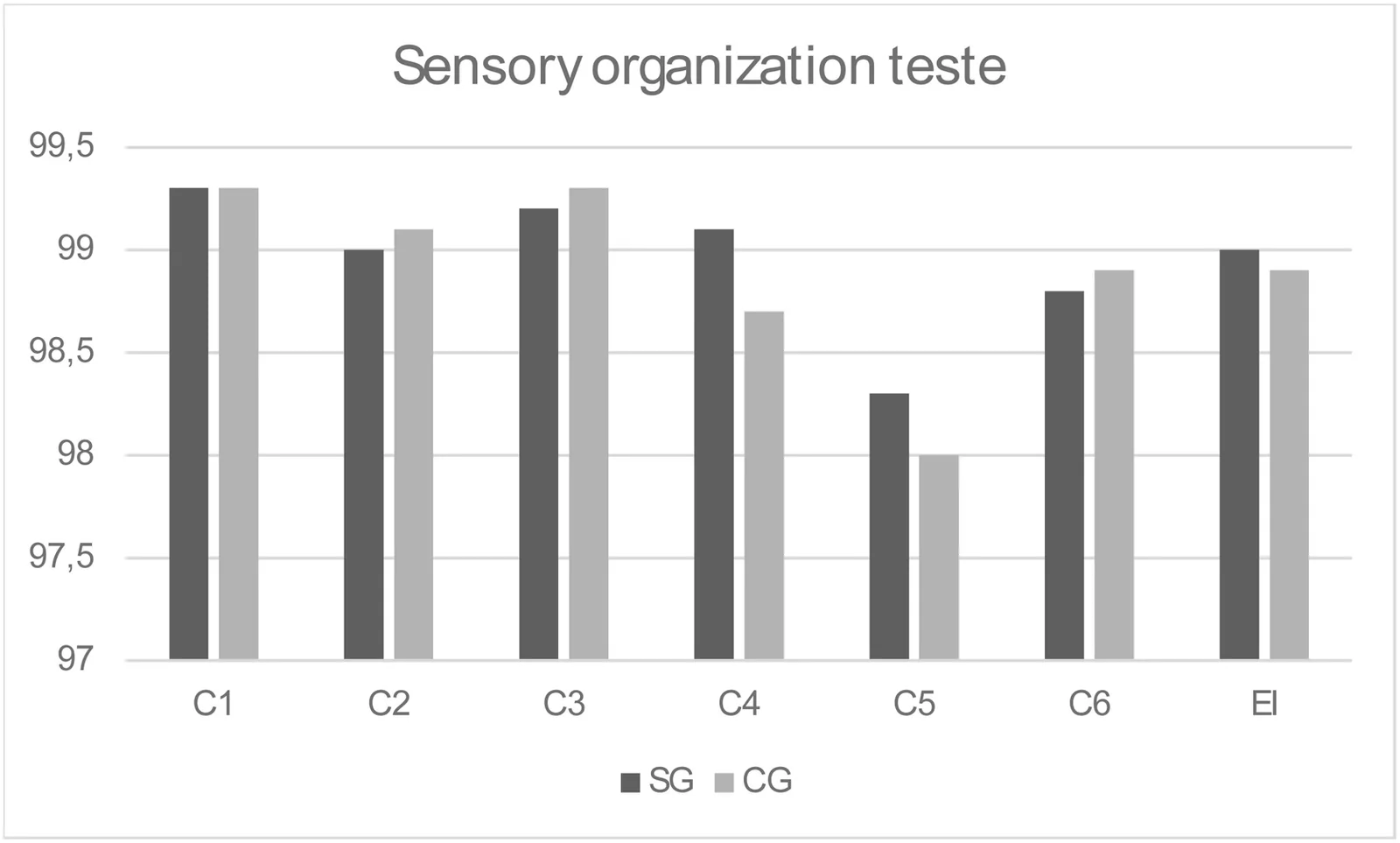
Scores for each of the sensory organization test conditions. and the composite score. C, Condition; EI, Equilibrium index; SG, Study Group; CG, Control Group.

**Fig. 3:**
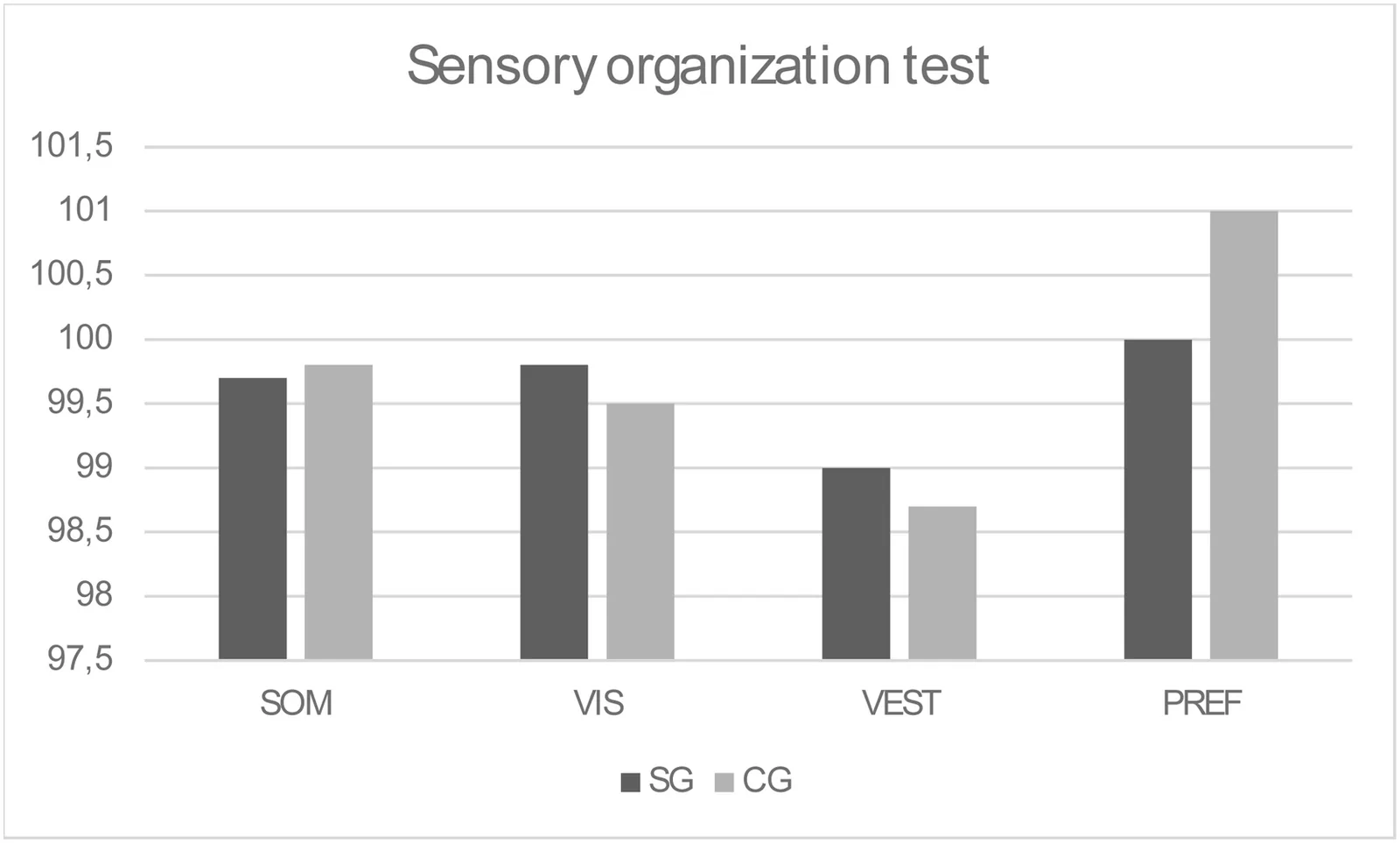
Scores for each of the sensory organization patterns. SOM, Somatossensory; VIS, Visual; VEST, Vestibular; PREF, Visual Preference; SG, Study Group; CG, Control Group.

## Discussion

Postural balance depends on sensory afferents, motor efferents, and integrative capacity. The Central Nervous System (CNS) receives inputs from the vestibular, visual, and proprioceptive systems and integrates them to construct a real-time model of body position and movement. This representation is compared to a pre-existing internal map of postural situations, and motor outputs are generated to adjust posture accordingly. When the incoming sensory information does not match the stored representations, a sensory conflict signal is generated, leading to the symptoms of motion sickness.^1^^,^^9^

In situations involving environmental movement, postural adaptations are employed to minimize sensory conflict. Since CDP evaluates sensory integration, it was expected that the control group would exhibit greater postural stability than the motion sickness group. However, our results revealed no statistically significant differences between the groups. This absence of difference is noteworthy, considering the pathophysiology of motion sickness, where a manifestation of sensory conflict during testing was anticipated. Several factors may account for this, including the complexity of the postural control system and the possibility of habituation. Individuals with motion sickness remain constantly alert to environmental changes and must continually develop new strategies to maintain balance in different contexts.^5^^,^^10^ Moreover, repeated exposure to diverse stimuli during daily activities facilitates gradual updating of the internal postural representation, ultimately leading to concordance between actual and expected sensory inputs and resolution of symptoms. The youth and absence of vestibular disorders among our patients may have favored the development of effective habituation.^2^

Another important point is that motion sickness is a transient condition, and our results suggest that individuals with motion sickness do not demonstrate significant impairment of visual-vestibular integration for postural control outside of symptomatic episodes.

Currently, it is understood that although canal and otolith afferents are critical in symptom generation, the Velocity Storage Integrator (VSI) plays an essential role in activating the autonomic nervous system. The VSI is a neural network that involves the vestibular nuclei, the nucleus prepositus hypoglossi, the brainstem, and the nodulus and uvula of the cerebellum. This mechanism allows vestibular responses to persist beyond the cessation of the stimulus. Information from the semicircular canals charges the VSI network, sustaining the response through slow discharges of these neurons. There is also evidence that the VSI receives otolithic, visual, and proprioceptive inputs and projects to other neural complexes beyond ocular motor control. Thus, the velocity storage network operates in three dimensions, ensuring gaze stability as the head orientation changes relative to gravity.^11^^,^^12^

Motion sickness is mediated by the properties of the VSI, whose tendency is to align the ocular velocity generated by the angular vestibulo-ocular reflex with the Gravito-Inertial Acceleration (GIA). Therefore, disparity between the axis of eye rotation and the GIA, as well as the temporal properties of velocity storage (duration and intensity of the response), are crucial factors in triggering motion sickness symptoms. The nodulus and uvula of the vestibulocerebellum are likely the central sites where this disparity is detected, where vestibular time constants are habituated, and where autonomic connections responsible for symptoms are established.^13^, ^14^, ^15^

Thus, head movement relative to gravity is critical to the genesis of motion sickness, but symptom generation depends on VSI activity mediated by vestibular neurons in the superior and medial vestibular nuclei, under the control of the cerebellar nodulus. During posturography, there is no head movement, and therefore no activation of ampullary or otolithic signals. Consequently, the absence of stimuli to charge the VSI could explain the lack of alterations in sensory integration observed during posturography, as the typical sensory mismatch of motion sickness is not triggered.^14^^,^^15^ Additionally, posturography involves controlled and predictable stimuli, allowing balance systems to function appropriately even in individuals prone to motion sickness.

The postural control system must accommodate environmental changes. On a firm and stable surface, individuals primarily rely on somatosensory input for postural orientation.^16^ Our results show good performance of motion sickness subjects in conditions 1 and 2, which assess somatosensory input use, as well as in the somatosensory analysis overall.

When the support surface is unstable, visual and vestibular inputs become more critical. With eyes open, visual input predominates. Patients with motion sickness are expected to perform well in condition 4 and the visual analysis, given their preserved visual acuity and reliance on visual input for postural maintenance, as confirmed in our study.^16^

Vestibular input becomes crucial when visual and somatosensory information is unavailable or conflicting.^16^ Some studies have reported lower equilibrium scores in condition 5 and in vestibular analysis among motion sickness subjects, suggesting greater dependence on a stable surface and visual cues, consistent with relative vestibular dysfunction or decreased vestibular input use.^3^^,^^4^ However, our study did not demonstrate significant differences between groups in condition 5 or in vestibular analysis, suggesting preserved vestibular function among our motion sickness patients. Several authors have reported that individuals with vestibular impairment are less susceptible to motion sickness compared to those with preserved vestibular function, strongly suggesting vestibular involvement in motion sickness. With vestibular hypofunction, ampullary signaling is compromised, resulting in insufficient VSI charging and thus a relative immunity to motion sickness. Similar effects have been observed following lesions of the cerebellar nodulus and uvula, or with the use of baclofen, a GABAergic agonist that modulates the VSI system.^7^^,^^17^^,^^18^

Individuals with motion sickness tend to rely less on vestibular input and more on visual information to maintain balance.^4^^,^^5^ Alharbi et al. observed worse postural control in motion sickness subjects exposed to virtual reality stimuli compared to controls, independent of sex. Young adults with motion sickness exhibited greater postural sway, suggesting that postural stability in these individuals is more affected by deceptive visual stimuli.^5^ Whitney et al. suggested that sensitivity to visual disturbances and visual dependence develop when there is a preference for visual cues.^19^ It is likely that the CNS continues to rely on visual information even when it provides inaccurate references in individuals prone to motion sickness. On the other hand, Tal et al. demonstrated longitudinal habituation to sensory conflict in motion sickness-prone individuals. Initially, these individuals intensify reliance on visual and somatosensory inputs for postural control, developing motion sickness symptoms under conflicting conditions. With repeated exposure, they increasingly rely on vestibular cues and disregard misleading visual input, reflecting increased habituation to motion sickness⁶. It has also been shown that peripheral ocular stimuli have greater capacity to activate the VSI compared to stimuli presented in the central visual field.^15^

We did not observe worse performance in conditions 3 and 6 among motion sickness patients compared to controls, and we infer several possible explanations.

The equipment used in this study did not allow for the presentation of visual stimuli in the peripheral visual field. This may have been the main limiting factor of our study, as it did not provide a sufficiently robust visual input to induce sensory conflict, as typically observed in protocols employing virtual reality environments.^5^

Additionally, the sensation of rotation varies with VSI activity.^20^ As previously discussed, posturography maintains head alignment in the vertical plane, not stimulating the VSI. Finally, it is known that patients with vestibular migraine have abnormally elevated reflex and perceptual vestibular thresholds due to altered cortical visuo-vestibular interactions, resulting in greater stimulus tolerance.^21^ Given the well-established relationship between motion sickness and vestibular migraine,^22^^,^^23^ it is reasonable to hypothesize that patients with motion sickness may similarly exhibit habituation, explaining the absence of alterations under conflicting posturography conditions. As a result of this habituation, individuals learn to disregard misleading visual cues and rely more on vestibular inputs for balance control.

Vestibular information also contributes to another critical internal framework: the map of stability limits. Stability limits refer to the maximum safe leaning angles and vary by sex, age, and other factors.^8^ Abnormal vestibular input may impair the comparison between actual stability limits and the internal map.^1^^,^^9^ In our study, no significant difference in SLA was found between groups, likely due to the good vestibular function and favorable age range of participants.

Our study has limitations, particularly concerning the characteristics of the sample. Exclusion of individuals with vestibular disorders precluded the analysis of motion sickness and sensory conflict effects in patients with vestibular diseases. Additionally, the cross-sectional design, which assessed participants at a single time point, limited evaluation of a dynamic condition that may vary according to environmental stimuli. Furthermore, as this study was designed as an exploratory observational investigation, no *a priori* sample size calculation was performed, and the results should be interpreted with the understanding that small effect differences between groups may not have been detected.

Despite these limitations, the present findings provide important insights into the relationship between motion sickness and postural control. The absence of significant differences in posturographic performance between participants with motion sickness and healthy controls does not necessarily contradict the initial hypothesis but rather provides important insights into the nature of motion sickness and its relationship with postural control. Motion sickness is widely conceptualized as a context-dependent phenomenon arising from sensory conflict or neural mismatch, which typically manifests under dynamic and provocative sensory conditions rather than during stable, static tasks. Consequently, posturographic assessments performed under predictable and non-conflicting conditions may not be sufficiently sensitive to detect alterations associated with motion sickness susceptibility. In addition, individuals prone to motion sickness may develop effective compensatory strategies, such as sensory reweighting or increased postural stiffness, allowing them to maintain postural stability comparable to that of asymptomatic individuals. Thus, similar postural outcomes between groups may reflect different underlying control strategies rather than identical sensorimotor processing. Finally, the episodic nature of motion sickness and the heterogeneity of susceptibility within the affected group may further contribute to the lack of observable differences in conventional posturographic metrics.

## Conclusion

Individuals with motion sickness demonstrated posturographic performance comparable to that of asymptomatic controls under the conditions evaluated in this study. These findings indicate that motion sickness susceptibility is not necessarily associated with impaired baseline postural stability, but rather with context-dependent alterations in sensory integration that may only become evident under provocative or dynamic sensory conditions. This study contributes to a more nuanced understanding of the relationship between motion sickness and postural control and underscores the importance of task demands and sensory context when interpreting posturographic outcomes in this population.

## ORCID ID

Arthur Jose Roque Cruz: 0000-0002-8576-9630

Paola Piva de Freitas: 0000-0002-1027-8415

Danilo Costa Chagas: 0009-0004-2658-4574

Raquel Mezzalira: 0000-0002-0279-8007

Maria Isabel Ramos do Amaral: 0000-0002-6909-3597

Carlos Takahiro Chone: 0000-0002-4217-4629

## Funding

None.

## Data availability statement

The authors declare that all data are available in repository.

## Declaration of competing interest

The authors declare no conflicts of interest.
